# Supplementary material for: The Caenorhabditis elegans Tubby homolog dynamically modulates olfactory cilia membrane morphogenesis and phospholipid composition
Source: eLife. 2019 Jul 1;8:e48789. doi: 10.7554/eLife.48789 (PMC6624019; doi:10.7554/eLife.48789)
Supplement: Supplementary file 1. [file elife-48789-supp1.docx]

**Supplementary File 1.** List of strains used in this work.

| **Strain** | **Genotype** | **Source** |
| --- | --- | --- |
| PY10210 | *oyIs88[gpa-4Δ6*p*::myr-gfp]* V | From A. Maurya |
| PY10211 | *tub-1(nr2004)* II*;* *oyIs88[gpa-4Δ6*p*::myr-gfp]* V | This work |
| PY3453 | *oyIs50[ceh-36*p*::gfp]* IV | (Kim et al., 2010) |
| PY10205 | *tub-1(nr2004)* II*; oyIs50[ceh-36*p*::gfp]* IV | This work |
| PY1089 | *kyIs104[str-1*p*::gfp]* X | (Troemel et al., 1997) |
| PY10200 | *tub-1(nr2004)* II*; kyIs104[str-1*p*::gfp]* X | This work |
| PY10212 | *tub-1(nr2044)* II*; kyIs104[str-1*p*::gfp]* X | This work |
| PY10213 | *tub-1(nr2004)* II*; kyIs104[str-1*p*::gfp]* X*; oyEx611[str-1*p*::tagRfp::tub-1, unc-122*p*::dsRed]* | This work |
| PY10214 | *oyEx600[str-1*p*::osm-6::gfp, str-1*p*::myr-tagRfp, unc-122*p*::gfp]* | This work |
| PY10215 | *tub-1(nr2004)* II*; oyEx600[str-1*p*::osm-6::gfp, str-1*p*::myr-tagRfp, unc-122*p*::gfp]* | This work |
| PY1058 | *oyIs14[sra-6*p*::gfp]* V | (Troemel et al., 1997) |
| PY10201 | *tub-1(nr2004)* II*; oyIs14[sra-6*p*::gfp]* V | This work |
| PY8815 | *Ex[srg-47*p*::gfp, unc-122*p*::dsRed]* | (McGrath et al., 2011; Nechipurenko et al., 2016) |
| PY10203 | *tub-1(nr2004)* II; *Ex[srg-47*p*::gfp, unc-122*p*::dsRed]* | This work |
| PY10216 | *oyEx612[sra-9*p*::myr-gfp, unc-122*p*::gfp]* | This work |
| PY10217 | *tub-1(nr2004)* II*; oyEx612[sra-9*p*::myr-gfp, unc-122*p*::gfp]* | This work |
| PY7306 | *oyIs65[str-1*p*::mCherry]* | (Nechipurenko et al., 2016) |
| PY10218 | *tub-1(nr2004)* II*;* *oyIs65[str-1*p*::mCherry]* | This work |
| PY10219 | *tub-1(nr2004)* II*;* *oyIs65[str-1*p*::mCherry]; oyEx613[str-1*p*::gfp::HsTulp1, unc-122*p*::gfp]* | This work; S. Mukhopadhyay |
| PY10220 | *tub-1(nr2004)* II*;* *oyIs65[str-1*p*::mCherry]; oyEx614[str-1*p*::gfp::HsTulp3, unc-122*p*::gfp]* | This work; S. Mukhopadhyay |
| PY8169 | *oyIs65[str-1*p*::mCherry]; Ex[str-1*p*::tax-4::gfp, unc-122*p*::gfp]* | (Wojtyniak et al., 2013) |
| PY10221 | *odr-3(Q206L)XS* V*; Ex[str-1*p*::tax-4::gfp, unc-122*p*::gfp]* | This work; (Roayaie et al., 1998) |
| PY10222 | *oyEx615[str-1*p*::srbc-64::gfp, str-1*p*::tagRfp, unc-122*p*::gfp]* | This work |
| PY10223 | *tub-1(nr2004)* II*;* *oyEx615[str-1*p*::srbc-64::gfp, str-1*p*::Rfp, unc-122*p*::gfp]* | This work |
| PY10224 | *tub-1(nr2004)* II*;* *oyIs65[str-1*p*::mCherry]; Ex[str-1*p*::tax-4::gfp, unc-122*p*::gfp]* | This work |
| PY10225 | *kyIs104[str-1*p*::gfp]* X*; oyEx601[str-1*p*::arl-13::tagRfp, unc-122*p*::gfp]* | This work |
| PY10226 | *tub-1(nr2004)* II*;* *kyIs104[str-1*p*::gfp]* X*; oyEx601[str-1*p*::arl-13::tagRfp, unc-122*p*::gfp]* | This work |
| PY9420 | *oyIs65[str-1*p*::mCherry]; Ex[str-1*p*::dyf-19::gfp, unc-122*p*::gfp]* | (Nechipurenko et al., 2016) |
| PY10227 | *tub-1(nr2004)* II*; oyIs65[str-1*p*::mCherry]; Ex[str-1*p*::dyf-19::gfp, unc-122*p*::gfp]* | This work |
| PY8859 | *oyIs65[str-1*p*::mCherry]; Ex[str-1*p*::mks-5::gfp, unc-122*p*::gfp]* | (Nechipurenko et al., 2016) |
| PY10228 | *tub-1(nr2004)* II*; oyIs65[str-1*p*::mCherry]; Ex[str-1*p*::mks-5::gfp, unc-122*p*::gfp]* | This work |
| PY8181 | *Ex[srbc-66*p*::tax-2::gfp, srbc-66*p*::che-13::tagRfp, unc-122*p*::gfp]* | (Wojtyniak et al., 2013) |
| PY10229 | *tub-1(nr2004)* II*;* *Ex[srbc-66*p*::tax-2::gfp, srbc-66*p*::che-13::tagRfp, unc-122*p*::gfp]* | This work |
| PY10230 | *oyIs14[sra-6*p*::gfp]* V*; oyEx602[sra-6*p*::arl-13::tagRfp, unc-122*p*::gfp]* | This work |
| PY10231 | *tub-1(nr2004)* II*; oyIs14[sra-6*p*::gfp]* V*; oyEx602[sra-6*p*::arl-13::tagRfp, unc-122*p*::gfp]* | This work |
| PY10232 | *tub-1(nr2004)* II*; kyIs104[str-1*p*::gfp]* X*; oyEx616[str-1*p*::tagRfp::tub-1(aa1-164), unc-122*p*::dsRed]* | This work |
| PY10233 | *tub-1(nr2004)* II*; kyIs104[str-1*p*::gfp]* X*; oyEx617[str-1*p*::tagRfp::tub-1(K250A, R251A), unc-122*p*::dsRed]* | This work |
| PY10234 | *daf-10(e1387)* IV*; kyIs104[str-1*p*::gfp]* X | This work |
| PY10235 | *oyIs65[str-1*p*::mCherry];* *Ex[str-1*p*::daf-10::gfp, unc-122*p*::dsRed]* | This work |
| PY10236 | *tub-1(nr2004)* II*; oyIs65[str-1*p*::mCherry];* *Ex[str-1*p*::daf-10::gfp, unc-122*p*::dsRed]* | This work |
| PY10237 | *odr-1(n1936)* X*; kyIs104[str-1*p*::gfp]* X | This work |
| PY10238 | *daf-10(e1387)* IV*; odr-1(n1936)* X*; kyIs104[str-1*p*::gfp]* X | This work |
| PY10239 | *tub-1(nr2004)* II*; kyIs104[str-1*p*::gfp]* X*; oyEx618[str-1*p*::tagRfp::tub-1(aa165-426), unc-122*p*::dsRed]* | This work |
| PY10240 | *tub-1(nr2004)* II*; kyIs104[str-1*p*::gfp]* X*; oyEx603[str-1*p*::tagRfp::tub-1(Q16A, R17A, K27A, R28A), unc-122*p*::gfp]* | This work |
| PY10280 | *tub-1(nr2004)* II*; kyIs104[str-1*p*::gfp]* X*; oyEx629[str-1*p*::tagRfp::tub-1(Q16A, R17A, K27A, R28A), unc-122*p*::gfp]* | This work |
| PY10241 | *tub-1(nr2004)* II*; kap-1(ok676)* III*; kyIs104[str-1*p*::gfp]* X*; oyEx611[str-1*p*::tagRfp::tub-1, unc-122*p*::dsRed]* | This work |
| PY10242 | *inpp-1(gk3262)* IV*; oyIs65[str-1*p*::mCherry]; oyEx604[str-1*p*::inpp-1a::gfp, unc-122*p*::gfp]* | This work |
| PY10243 | *tub-1(nr2004)* II*; inpp-1(gk3262)* IV*; oyIs65[str-1*p*::mCherry]; oyEx604[str-1*p*::inpp-1a::gfp, unc-122*p*::gfp]* | This work |
| PY10244 | *inpp-1(gk3262)* IV*; odr-1(n1936)* X*; oyIs65[str-1*p*::mCherry]; oyEx604[str-1*p*::inpp-1a::gfp, unc-122*p*::gfp]* | This work |
| PY10245 | *tub-1(nr2004)* II*; odr-1(n1936)* X*; oyEx619[str-1*p*::myr-gfp, unc-122*p*::gfp]* | This work |
| PY10246 | *odr-1(n1936)* X*; oyEx619[str-1*p*::myr-gfp, unc-122*p*::gfp]* | This work |
| PY10247 | *tub-1(nr2004)* II*; oyEx619[str-1*p*::myr-gfp, unc-122*p*::gfp]* | This work |
| PY10248 | *oyEx619[str-1*p*::myr-gfp, unc-122*p*::gfp]* | This work |
| PY10249 | *tub-1(nr2004)* II*; oyEx620[srd-23*p*::gfp::tub-1::SL2::mScarlet, unc-122*p*::gfp]* | This work |
| PY10250 | *tub-1(nr2004)* II*; odr-1(n1936)* X*; oyEx621[srd-23*p*::gfp::tub-1::SL2::mScarlet, unc-122*p*::gfp]* | This work |
| PY10251 | *oyEx622[srd-23*p*::gfp::PLCδ1-PH::SL2::mScarlet, unc-122*p*::gfp]* | This work |
| PY10252 | *tub-1(nr2004)* II*; oyEx622[srd-23*p*::gfp::PLCδ1-PH::SL2::mScarlet, unc-122*p*::gfp]* | This work |
| PY10253 | *tub-1(nr2004)* II*; oyEx622[srd-23*p*::gfp::PLCδ1-PH::SL2:mScarlet, unc-122*p*::gfp]; oyEx611[str-1*p*::tagRfp::tub-1, unc-122*p*::dsRed]* | This work |
| PY10254 | *odr-1(n1936)* X*; oyEx623[srd-23*p*::gfp::PLCδ1-PH::SL2::mScarlet, unc-122*p*::gfp]* | This work |
| PY10255 | *inpp-1(gk3262)* IV*; oyEx622[srd-23*p*::gfp::PLCδ1-PH::SL2::mScarlet, unc-122*p*::gfp]* | This work |
| PY10256 | *kyIs104[str-1*p*::gfp]* X*; oyEx611[str-1*p*::tagRfp::tub-1, unc-122*p*::dsRed]* | This work |
| PY10257 | *inpp-1(gk3262)* IV*; kyIs104[str-1*p*::gfp]* X*; oyEx611[str-1*p*::tagRfp::tub-1, unc-122*p*::dsRed]* | This work |
| PY10258 | *inpp-1(gk262); kyIs104[str-1*p*::gfp]* X*; oyEx611[str-1*p*::tagRfp::tub-1, unc-122*p*::dsRed]; oyEx604[str-1*p*::inpp-1a::gfp, unc-122*p*::gfp]* | This work |
| PY10259 | *oyEx605[srd-23*p*::gfp::ppk-1::SL2::mScarlet, unc-122*p*::gfp]* | This work |
| PY10260 | *odr-1(n1936)* X*; oyEx606[srd-23*p*::gfp::ppk-1::SL2::mScarlet, unc-122*p*::gfp]* | This work |
| PY10261 | *oyEx624[sra-9*p*::gfp::tub-1::SL2::mScarlet, unc-122*p*::gfp]* | This work |
| PY10262 | *oyEx625[sra-9*p*::gfp::PLCδ1-PH::SL2::mScarlet, unc-122*p*::gfp]* | This work |
| PY10263 | *tub-1(nr2004)* II*; oyEx625[sra-9*p*::gfp::PLCδ1-PH::SL2::mScarlet, unc-122*p*::gfp]* | This work |
| PY10264 | *oyEx626[sra-9*p*::mScarlet, unc-122*p*::mCherry]; oyEx627[sra-9*p*::gfp::ppk-1, unc-122*p*::gfp]* | This work |
| PY10265 | *tub-1(nr2004)* II*; oyEx626[sra-9*p*::mScarlet, unc-122*p*::mCherry]; oyEx627[sra-9*p*::gfp::ppk-1, unc-122*p*::gfp]* | This work |
| PY10266 | *tub-1(nr2004)* II*; oyEx605[srd-23*p*::gfp::ppk-1::SL2::mScarlet, unc-122*p*::gfp]* | This work |
| PY10267 | *tub-1(nr2004)* II*; odr-1(n1936)* X*; oyEx606[srd-23*p*::gfp::ppk-1::SL2::mScarlet, unc-122*p*::gfp]* | This work |
| PY10268 | *tub-1(nr2004)* II*; odr-1(n1936)* X*; oyEx623[srd-23*p*::gfp::PLCδ1-PH::SL2::mScarlet, unc-122*p*::gfp]* | This work |
| PY10269 | *oyIs65[str-1*p*::mCherry]; oyEx607[str-1*p*::dyn-1::gfp, unc-122*p*::gfp]* | This work |
| PY10270 | *tub-1(nr2004)* II*; oyIs65[str-1*p*::mCherry]; oyEx607[str-1*p*::dyn-1::gfp, unc-122*p*::gfp]* | This work |
| PY10271 | *oyEx626[sra-9*p*::mScarlet, unc-122*p*::mCherry]; oyEx608[sra-9*p*::dpy-23::gfp, unc-122*p*::gfp]* | This work |
| PY10272 | *tub-1(nr2004)* II*; oyEx626[sra-9*p*::mScarlet, unc-122*p*::mCherry]; oyEx608[sra-9*p*::dpy-23::gfp, unc-122*p*::gfp]* | This work |
| PY10273 | *oyEx626[sra-9*p*::mScarlet, unc-122*p*::mCherry]; oyEx609[sra-9*p*::dyn-1::gfp, unc-122*p*::gfp]* | This work |
| PY10274 | *tub-1(nr2004)* II*; oyEx626[sra-9*p*::mScarlet, unc-122*p*::mCherry]; oyEx609[sra-9*p*::dyn-1::gfp, unc-122*p*::gfp]* | This work |
| PY10275 | *oyIs65[str-1*p*::mCherry]; oyEx610[str-1*p*::dpy-23::gfp, unc-122*p*::gfp]* | This work |
| PY10276 | *tub-1(nr2004)* II*; oyIs65[str-1*p*::mCherry]; oyEx610[str-1*p*::dpy-23::gfp, unc-122*p*::gfp]* | This work |
| PY10277 | *dpy-23(e840)* X*; oyEx622[srd-23*p*::gfp::PLCδ1-PH::SL2::mScarlet, unc-122*p*::gfp]* | This work |
| PY12000 | *dpy-23(e840)* X*; kyIs104[str-1*p*::gfp]* X | This work |
| PY10278 | *tub-1(nr2004)* II*; dpy-23(e840)* X*; kyIs104[str-1*p*::gfp]* X | This work |
| PY10279 | *tub-1(nr2004)* II*; dpy-23(e840)* X*; kyIs104[str-1*p*::gfp]* X*; oyEx611[str-1*p*::tagRfp::tub-1, unc-122*p*::dsRed]* | This work |

**REFERENCES**

Kim, K., Kim, R., and Sengupta, P. (2010). The HMX/NKX homeodomain protein MLS- 2

specifies the identity of the AWC sensory neuron type via regulation of the *ceh- 36 Otx*

gene in *C. elegans*. Development *137*, 963-974.

McGrath, P.T., Xu, Y., Ailion, M., Garrison, J.L., Butcher, R.A., and Bargmann, C.I. (2011).

Parallel evolution of domesticated *Caenorhabditis* species targets pheromone receptor

genes. Nature *477*, 321-325.

Nechipurenko, I.V., Olivier-Mason, A., Kazatskaya, A., Kennedy, J., McLachlan, I.G., Heiman,

M., Blacque, O.E., and Sengupta, P. (2016). A conserved role for Girdin in basal body

positioning and ciliogenesis. Dev Cell *38*, 493-506.
