# Supplementary material for: The Caenorhabditis elegans Tubby homolog dynamically modulates olfactory cilia membrane morphogenesis and phospholipid composition
Source: eLife. 2019 Jul 1;8:e48789. doi: 10.7554/eLife.48789 (PMC6624019; doi:10.7554/eLife.48789)
Supplement: Supplementary file 2. [file elife-48789-supp2.docx]

**Supplementary File 2.** List of plasmids used in this work.

| **Plasmid** | **Description** | **Source** |
| --- | --- | --- |
| PSAB1120 | *gpa-4Δ6*p::*myr-gfp* | (Maurya et al., 2019) |
| PSAB595 | *str-1*p::*osm-6::gfp* | (Mukhopadhyay et al., 2007) |
| PSAB1015 | *str-1*p::*arl-13::tagrfp* | (Nechipurenko et al., 2016) |
| PSAB1147 | *str-1*p::*myr-tagrfp* | This work |
| PSAB1148 | *sra-6*p::*arl-13::tagrfp* | This work |
| PSAB1149 | *str-1*p::*tagRFP::tub-1(Q16A, R17A, K27A, R28A)* | This work |
| PSAB1150 | *srd-23*p::*gfp::ppk-1::SL2::mScarlet* | This work |
| PSAB1151 | *str-1*p::*dyn-1::gfp* | This work |
| PSAB1152 | *sra-9*p::*dpy-23::gfp* | This work |
| PSAB1153 | *sra-9*p::*dyn-1::gfp* | This work |
| PSAB1154 | *str-1*p::*dpy-23::gfp* | This work |
| PSAB1155 | *str-1*p::*tagrfp::tub-1* | This work |
| PSAB1156 | *sra-9*p::*myr-gfp* | This work |
| PSAB1157 | *str-1*p::*gfp::tulp1* | This work |
| PSAB1158 | *str-1*p::*gfp::tulp3* | This work |
| PSAB1159 | *str-1*p::*srbc-64::gfp* | This work |
| PSAB1160 | *str-1*p::*tagrfp* | This work |
| PSAB1161 | *str-1*p::*tagrfp::tub-1(aa1-164)* | This work |
| PSAB1162 | *str-1*p::*tagrfp::tub-1(K250A, R251A)* | This work |
| PSAB1163 | *str-1*p::*tagrfp::tub-1(aa165-426)* | This work |
| PSAB1164 | *str-1*p::*inpp-1a::gfp* | This work |
| PSAB1165 | *str-1*p::*myr-gfp* | This work |
| PSAB1166 | *srd-23*p::*gfp::tub-1::SL2::mScarlet* | This work |
| PSAB1167 | *srd-23*p::*gfp::PLCδ1-PH::SL2::mScarlet* | This work |
| PSAB1168 | *sra-9*p::*gfp::tub-1::SL2::mScarlet* | This work |
| PSAB1169 | *sra-9*p::*gfp*::*PLCδ1-PH::SL2::mScarlet* | This work |
| PSAB1170 | *sra-9*p::*mScarlet* | This work |
| PSAB1171 | *sra-9*p::*gfp::ppk-1* | This work |

**REFERENCES**

Maurya, A.K., Rogers, T., and Sengupta, P. (2019). A CCRK and a MAK kinase modulate cilia

branching and length via regulation of axonemal microtubule dynamics in

*Caenorhabditis elegans*. Curr Biol *22*, 1286-1300.

Nechipurenko, I.V., Olivier-Mason, A., Kazatskaya, A., Kennedy, J., McLachlan, I.G., Heiman,

M., Blacque, O.E., and Sengupta, P. (2016). A conserved role for Girdin in basal body

positioning and ciliogenesis. Dev Cell *38*, 493-506.
